# Intraneural Topography of Rat Sciatic Axons: Implications for Polyethylene Glycol Fusion Peripheral Nerve Repair

**DOI:** 10.3389/fncel.2022.852933

**Published:** 2022-03-30

**Authors:** Emily A. Hibbard, Dale R. Sengelaub

**Affiliations:** Department of Psychological and Brain Sciences, Indiana University, Bloomington, IN, United States

**Keywords:** sciatic nerve, topography, rat, retrograde labeling, morphology

## Abstract

Peripheral nerve injuries are the most common type of nerve trauma. We have been working with a novel repair technique using a plasmalemmal fusogen, polyethylene glycol (PEG), to re-fuse the membranes of severed axons. PEG-fusion repair allows for immediate re-innervation of distal targets, prevents axonal degeneration, and improves behavioral recovery. PEG-fusion of severed axons is non-specific, and we have previously reported that following injury and PEG-fusion misconnections between spinal motoneurons and their distal targets were present. Surprisingly, appropriately paired proximal and distal motor axons were observed in all PEG-fused animals. We hypothesized that a topographic organization of axons contributing to the sciatic nerve could explain the incidence of appropriate connections. We traced the course of specific axon populations contributing to the sciatic nerve in young adult male and female rats. Following intraneural injection of Fast Blue into the tibial branch, labeled axons were confined to a discrete location throughout the course of the nerve. Following intramuscular injection of cholera toxin-conjugated horseradish peroxidase into the anterior tibialis, labeled axons were confined to a smaller but still discrete location throughout the nerve. In both cases, the relative locations of labeled axons were consistent bilaterally within animals, as well as across animals and sexes. Thus, the relatively consistent location of specific axon populations could allow for realignment of appropriate populations of axons, and enhanced behavioral recovery seen in PEG-fused animals. Knowing the organization of axons within the sciatic nerve promotes accurate territory realignment during repair, therefore aiding in recovery outcomes.

## Introduction

Peripheral nerve injuries (PNIs) that disrupt axonal continuity, therefore denervating distal musculature and tissue, have serious consequences on both the peripheral and central nervous systems. The peripheral consequences include the immediate loss of both motor and sensory function distal to the injury, rapid and irreversible degeneration of the distal nerve segment (Wallerian degeneration), as well as neuromuscular junction loss and muscle atrophy over time (Brushart, 2011). Centrally, PNIs lead to motoneuron somal atrophy (Ma et al., 2002; Wiberg et al., 2017), retraction of synaptic inputs from somata and dendrites (Alvarez et al., 2011; Rotterman et al., 2014; Wiberg et al., 2017), and dendritic atrophy (Rotterman et al., 2014; Wiberg et al., 2017). Reinnervation of distal targets depends upon the slow outgrowth of axons from the proximal nerve stump which may take several weeks to reestablish any connectivity (Wolfe et al., 2010). The current standard of treatment for PNIs is neurorrhaphy which involves microsuturing the epineuria of the proximal and distal severed nerve stumps (Brushart, 2011). Unfortunately, this leaves internal axons in their severed state, does not prevent Wallerian degeneration, and only marginally improves recovery (Allen, 2000; Bertelli and Ghizoni, 2009, 2011). Overall, behavioral recovery, with and without standard treatment, is very poor, which reinforces the need for a new treatment protocol for PNIs.

A novel repair technique for PNIs called polyethylene glycol (PEG) fusion repair has shown tremendous therapeutic potential. PEG-fusion uses a plasmalemmal fusogen, PEG, to fuse severed axonal membranes after injury thus immediately restoring axonal continuity and distal innervation. PEG-fusion repair improves behavioral recovery remarkably, and has been shown to prevent both the peripheral and central consequences of PNI including Wallerian degeneration (Britt et al., 2010; Riley et al., 2015; Ghergherehchi et al., 2016; Mikesh et al., 2018a,b) and somal/dendritic atrophy (Ghergherehchi et al., 2019). Fusion of severed axonal membranes by PEG-fusion is non-specific; therefore, PEG-fusion indiscriminately fuses axonal membranes that are in close approximation without regard for their modality or original connectivity. We have previously reported that this non-specificity leads to misconnections between motoneurons and their distal targets (Ghergherehchi et al., 2019). After sciatic nerve injury and PEG-fusion, we performed an intramuscular injection of retrograde tracer into the anterior tibialis (TA) muscle in order to label innervating motoneurons, and found labeled motoneurons in inappropriate spinal segments, indicating misconnections (Ghergherehchi et al., 2019). Surprisingly, labeled motoneurons were also found in the appropriate spinal segment in every PEG-fused animal, regardless of repair type, either single-cut or allograft (Ghergherehchi et al., 2019). Despite PEG-fusion non-specifically fusing severed axons, proximal TA motor axons were reliably fused to distal axons that projected to TA.

We hypothesized that the consistent pattern of correctly connected TA axons after PEG-fusion repair was due to a topographic organization of axons within the sciatic nerve. It has been previously reported in a variety of species that axons which serve specific distal branches of peripheral nerves travel in organized bundles along the course of the nerve (Sunderland, 1978; Brushart, 1991; Hallin et al., 1991). The sciatic nerve of rats has been shown to exhibit a similar organization of its major branches (Suaid et al., 2016; Ravagli et al., 2020) as well as its more distal branches (Badia et al., 2010). We investigated the topographic organization of axons within the rat sciatic nerve at the level of an individual muscle (TA), and looked for consistencies in this topography bilaterally within animals as well as across animals and sexes.

## Materials and Methods

All procedures were performed in accordance with the Indiana University Animal Care and Use Guidelines (Protocol #20-038). A total of 14 adult Sprague-Dawley rats (approximately 70 days old; Envigo, Indianapolis) were used (male, *n* = 8; female, *n* = 6). Rats were maintained on a 12:12-hr light/dark cycle with unlimited access to food and water.

### Tracer Injections

Intraneural labeling*:* The location of an axon population of a specific branch of the sciatic nerve was examined in a total of 12 nerves in adult male (*n* = 4) and female (*n* = 3) rats. Rats were anesthetized with isoflurane, and the sciatic nerve trifurcation was exposed along the dorsolateral aspect of the hindlimb. Just distal to the trifurcation, the tibial branch was isolated and crushed for ten seconds using atraumatic forceps in order to encourage uptake of the tracer. Using a Hamilton syringe connected to a 31G needle, 2 μL of Fast Blue (2.5%; Polysciences, Inc., Warrington, PA, United States) was injected into the tibial branch just proximal to the crush injury. Fast Blue is a fluorescent tracer that is retrogradely transported by motor and sensory axons (Kuypers and Huisman, 1984). Dissected muscles were sutured, and the skin incision was closed with wound clips. The same procedure was performed on the opposite hindlimb, and the animal was allowed to recover for six days, a period that ensures optimal transport (Steward, 1981).

Intramuscular labeling: The location of an axon population innervating a specific muscle was examined in a total of 14 sciatic nerves in adult male (*n* = 4) and female (*n* = 3) rats. Rats were anesthetized as above, and the anterior tibialis muscle was exposed and injected with horseradish peroxidase conjugated to the beta subunit of the cholera toxin molecule (BHRP; 2 μl, 0.2%; Invitrogen, Carlsbad, CA, United States). At this volume and concentration, BHRP is specifically taken up by motor axons and does not label dorsal root ganglion cells that lack GM1 ganglioside required to actively transmembrane transport BHRP (Alisky et al., 2002; Lappi et al., 2014). The skin incision was closed as above, and the animals were allowed to recover for two days, a period that ensures optimal transport (Kurz et al., 1986, 1991; Goldstein et al., 1990).

### Anatomical Dissection

All animals for both injection groups followed the same dissection methodology. After the appropriate transport period, animals were weighed, given an overdose of urethane (approximately 0.5g/100g body weight), and perfused intracardially with saline followed by cold fixative (4% paraformaldehyde). The entirety of the sciatic nerve was exposed along the dorsolateral aspect of the hindlimb by blunt dissection of the muscles from the sciatic notch to the distal trifurcation. Two 10 mm sciatic nerve segments were removed, a proximal segment starting at the sciatic notch and a distal segment terminating at the trifurcation. Heat lesions were placed on the lateral aspect of each segment to serve as fiduciary marks to maintain orientation of nerve segments throughout processing. The same dissection process was performed on the opposite hindlimb. The nerve segments were post-fixed in the same fixative for two hours, then transferred to 30% sucrose phosphate buffer overnight for cryoprotection.

### Histological Processing and Visualization

Nerve segments were embedded in a known orientation in M-1 Embedding Matrix (Shandon, Thermo Fisher Scientific Waltham, MA, United States) and sectioned transversely (50 μm, sampled at 500 μm intervals) on a cryostat at −15°C, thaw-mounted onto glass slides, and allowed to dry for 24 h.

For visualization of Fast Blue-labeled axons, sections were coverslipped with Vectashield antifade mounting medium with propidium iodide (Vector Laboratories, Inc., Burlingame, CA, United States). Sections spanning the length of the nerve segments (an average of 19.4 ± 1.4 sections per nerve segment) were observed under epifluorescence using a DAPI filter for visualization of Fast Blue and a Texas Red filter for visualization of propidium iodide. Digital micrographs of each section were taken at a final magnification of 166X using an NIS-Elements Imaging system (Nikon Instruments, Melville, NY, United States). Using the digital micrographs of propidium iodide-stained sections, the total nerve area was measured using a computer-based morphometry system (Stereo Investigator, MBF Bioscience, Williston, VT, United States). Using the matching digital micrographs of Fast Blue labeling, the area occupied and the distribution of labeled axons was mapped using the same computer-based morphometry system.

For visualization of BHRP-labeled axons, nerve sections were reacted using a modified tetramethyl benzidine protocol (Mesulam, 1982). Sections were counterstained with thionin and coverslipped with Permount (Fisher Scientific, Pittsburgh, PA, United States). Sections spanning the length of the nerve segments (an average of 19.2 ± 2.7 sections per nerve segment) were observed under darkfield illumination at a final magnification of 250X. The area and distribution of labeled axons within each section was reconstructed in three dimensions using a computer-based morphometry system (Neurolucida, MBF Bioscience). Digital light micrographs were obtained using an MDS 290 digital camera system (Eastman Kodak Company, Rochester, NY, United States). An analysis of variance with repeated measures was used to analyze the size of axon territories within the nerve.

## Results

### Fast Blue Labeling

**FIGURE 1:**
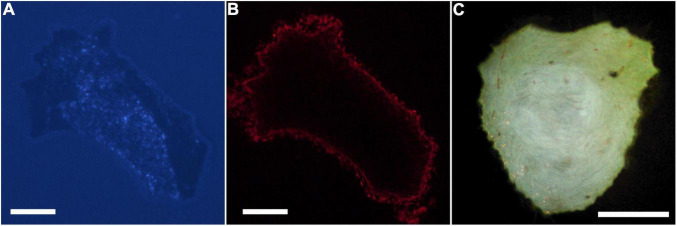
Digital micrographs of a transverse section of a proximal segment of a sciatic nerve visualizing. **(A)** Fast Blue-labeled axons contributing to the tibial branch (DAPI filter), and **(B)** the same section visualizing propidium iodide staining for total nerve area (Texas Red filter). **(C)** Darkfield digital micrograph of a transverse section of a proximal segment of a sciatic nerve showing BHRP-labeled TA motoneurons. Scale bars = 250 μm.

**FIGURE 2:**
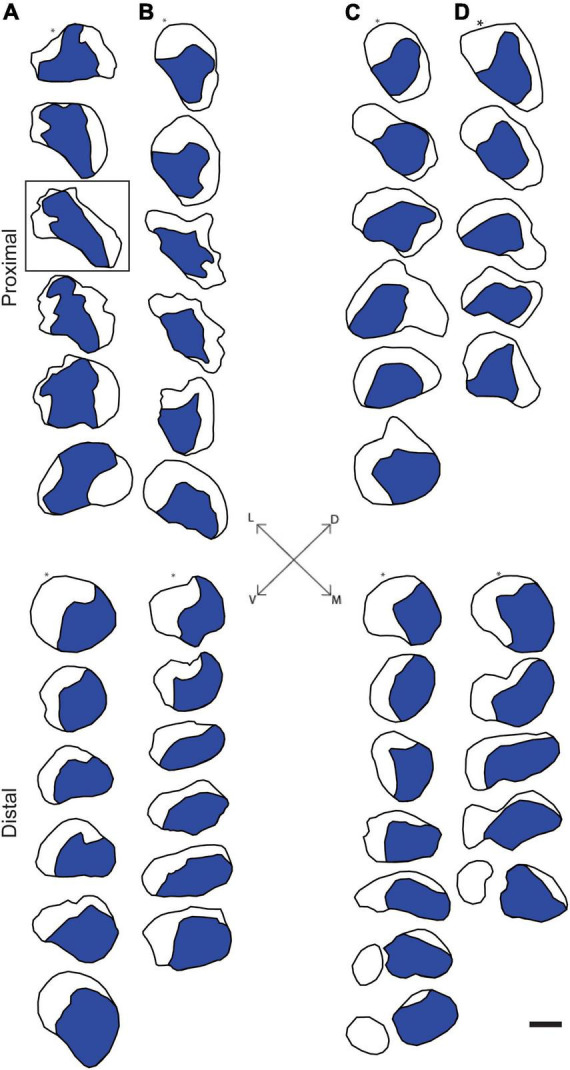
Representative reconstructions of the location of axons contributing to the tibial branch of the left **(A,C)** and right **(B,D)** sciatic nerves in a male **(A,B)** and a female **(C,D)** rat. Reconstructions were made at 500 μm intervals but are shown at 1,500 μm intervals for illustrative purposes. In all cases, Fast Blue-labeled axons (blue areas) were confined to a discrete location along the course of the sciatic nerve proximally from the sciatic notch to the distal trifurcation. Black outlines indicate the transverse area of the entire nerve measured from propidium iodide-stained images. Fiduciary marks indicated by asterisks in each reconstruction. Inset: Dorsoventral and mediolateral directions. Box indicates reconstruction of the same section shown in Figures 1A,B. Scale bar = 500 μm.

Digital micrographs of transverse sciatic nerve sections visualizing Fast Blue-labeled axons (Figure 1A) and propidium iodide staining (Figure 1B) showed a discrete localization of labeled axons. Reconstructions of Fast Blue-labeled axons within the sciatic nerve revealed a clearly defined and reproducible topographic organization (Figure 2). Labeled axons were found in a discrete location within the nerve from the sciatic notch to the distal trifurcation, rather than widely distributed across the entire transverse area. The tibial branch was also determined to occupy a relatively large transverse area of the sciatic nerve at an average of 53.40 ± 3.79%. We also examined the proximal, middle, and distal portions of the entire nerve for evidence of consistency in the size of the area occupied by Fast Blue-labeled axons. The area occupied by Fast Blue-labeled axons in the proximal portion of the nerve was 0.276 ± 0.034 mm^2^ (mean ± SEM). This area did not differ along the proximo-distal course of the nerve (middle portion, 0.302 ± 0.034 mm^2^; distal portion, 0.281 ± 0.029 mm^2^) [*F* (2,20) = 2.95, *ns*]. The size and position of the tibial branch territory was consistent bilaterally within animals, as well as across animals and sexes.

### BHRP Labeling

**FIGURE 3:**
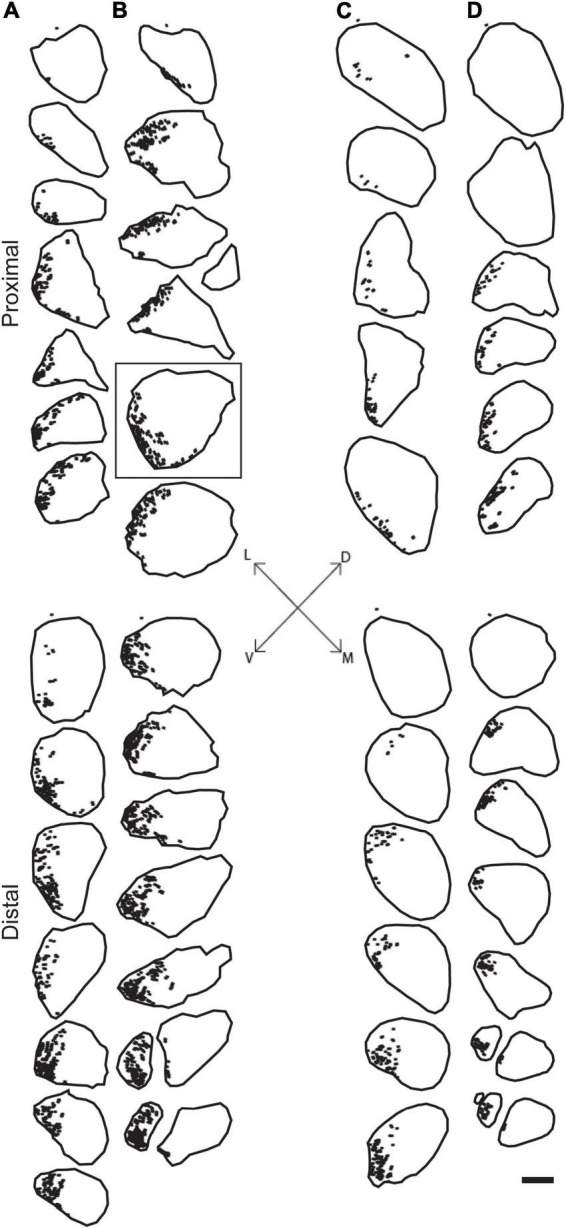
Representative reconstructions from the left **(A,C)** and right **(B,D)** sciatic nerves in a male **(A,B)** and female **(C,D)** rat showing the location of motor axons projecting to the TA muscle. Reconstructions were made at 500 μm intervals but are shown at 1,500 μm intervals for illustrative purposes. BHRP-labeled axons (indicated by black dots) were confined to a discrete position throughout the nerve. Black outlines indicate the transverse area of the entire nerve measured from darkfield images. Fiduciary marks indicated by asterisks in each reconstruction. Inset: Dorsoventral and mediolateral directions. Box indicates reconstruction of the same section shown in Figure 1C. Scale bar = 500 μm.

Digital micrographs of BHRP-labeled axons (Figure 1C) showed a discrete localization of labeled axons. Reconstructions of BHRP-labeled axons within the sciatic nerve (Figure 3) revealed a clearly defined and reproducible topographic organization (one nerve was excluded from further analysis due to compromised histology). Labeled axons were found in a discrete location throughout the entire nerve, rather than widely distributed across the entire transverse area. This territory was also relatively large, occupying 12.02 ± 1.01% of the total transverse area. The area occupied by BHRP-labeled axons in the proximal portion of the nerve was 0.035 ± 0.005 mm^2^. This area increased linearly along the proximo-distal course of the nerve to 0.047 ± 0.007 mm^2^ distally, an increase of 54.29% [*F* (2,22) = 17.92, *p* < 0.0001]. The size and position of the anterior tibialis territory was consistent bilaterally within animals, as well as across animals and sexes.

## Discussion

We assessed the distribution of axons contributing to either the tibial branch or the anterior tibialis muscle within the sciatic nerve. We found that both populations of axons occupy consistent and discrete territories within the nerve proximally from the sciatic notch to the distal trifurcation. The location and size of these territories was consistent bilaterally within animals as well as across animals and sexes.

Previous work has shown that specific populations of axons serving particular branches of a peripheral nerve run together along the course of the nerve. This theme has been replicated in a variety of species (humans, monkeys, rats), peripheral nerves (median, ulnar, radial, sciatic), and using a variety of techniques including anatomical dissection, retrograde labeling, microCT, and intrafascicular microstimulation. Sunderland’s work in human peripheral nerves demonstrated a somatotopic fascicular organization of axons within the distal portion of the nerve; however, this organization is lost in more proximal segments (Sunderland, 1978). Brushart’s work demonstrated the same distal organization in median nerves of monkeys, and confirmed its consistency more proximally using retrograde labeling techniques. Importantly, the position of these bundles of axons remained consistent throughout the nerve until the brachial plexus, and was consistent bilaterally within and across animals (Brushart, 1991). Previous work by Hallin et al. (1991) also confirmed intrafascicular organization of human peripheral nerves by showing sensory axons within the median nerve are segregated by sensory modality along the entirety of the nerve. More specifically, the human sciatic nerve also exhibits a somatotopic organization of intraneural fascicles as evidenced by magnetic resonance neurography of patients with spinal nerve root lesions (Bäumer et al., 2015).

The rat sciatic nerve exhibits a similar organization of specific axon populations. For instance, Suaid et al., 2016; Ravagli et al., 2020 both demonstrated that the axons contributing to the main branches of the sciatic (peroneal, tibial, and sural) stay discretely organized and do not intermingle for a significant distance proximal to the trifurcation. The same organization was found in the tibial branch of the sciatic nerve after retrogradely labeling individual distal branches contributing to small populations of muscles and tissue in the rat hindlimb (Badia et al., 2010). Our results are consistent with these previous findings: Tibial axons consistently occupied a discrete territory within the sciatic nerve proximally from the sciatic notch to the distal trifurcation. Importantly, these findings were consistent bilaterally within animals, as well as across animals and sexes.

**FIGURE 4:**
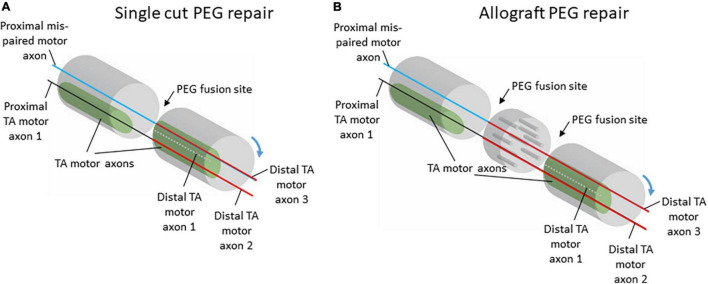
Schematic diagrams of potential proximal and distal axon fusing after single-cut **(A)** or allograft **(B)** repair with PEG-fusion. Although there is no expectation for specific re-pairing of severed axons with PEG-fusion, the relatively large and consistently located territory occupied by TA motor axons in the sciatic nerve allows for appropriate realignment of proximal and distal axon segments. Despite misalignment of axon populations through rotations of the nerve stumps during repair, the topography of TA motor axons would allow for some correct populations of proximal and distal axons to be fused.

The consistent organization of axons within the sciatic nerve could potentially explain the consistent pattern of anterior tibialis (TA) motoneuron labeling seen after PEG-fusion repair. The population of axons serving the TA muscle was reliably located in a consistent, discrete location along the length of the sciatic nerve. In addition to the consistency in location, the territory occupied by TA axons was also relatively large. These two features of the organization of TA axons within the sciatic nerve would allow for close approximation of severed proximal and distal TA axons during PEG-fusion repair (Figure 4). Therefore, proximal TA axons are extremely likely to be fused to distal axons projecting to TA, leading to the consistent pattern of appropriate connections induced after PEG-fusion repair seen in Ghergherehchi et al., 2019. Although these fusions are appropriate (i.e., proximal motor axons projecting to the TA are often fused to distal TA motor axons), there is no expectation that these fusions are rematches to the original axon segments.

In our previous work, the consistent pattern of appropriate connections to TA was present regardless of repair type, either single-cut or allograft repair (Ghergherehchi et al., 2019). For single-cut repairs, the organization of axons on the proximal stump will mirror the organization on the distal stump; therefore, territories should experience significant realignment even with the minimal, unavoidable rotations of the nerve stumps during repair (Figure 4). For allograft repairs, the same issue is relevant. Minimal rotations of the host nerve stumps should still leave large, consistent axon territories, like TA, mostly aligned. The allograft itself can be assumed to work similarly to a nerve conduit based on our results that show consistency in territory location along the course of the nerve (Figures 2, 3). Therefore, the allograft still allows for realignment of territories to be attained. Although appropriately fused TA axons were present regardless of repair type, the frequency of distal TA axons being fused to proximal axons from other populations of motoneurons was higher after allograft repairs (Ghergherehchi et al., 2019). This higher frequency of mispairing is likely due to there being two sites of PEG-fusion repair (one at each end of the donor graft) compared to the one site of PEG-fusion in single-cut repairs.

Reducing the frequency of mispairing through realignment of axon territories during repair may promote better behavioral recovery. Like rats, human peripheral nerves display a topographical organization (Sunderland, 1978; Bäumer et al., 2015), and thus realignment of axon territories within nerves should translate to humans. Successful PEG-fusion repair in human peripheral nerves has been reported. PEG-fusion repair of human digital nerves showed quick reestablishment of sensation as assessed by static two-point discrimination (Bamba et al., 2016). Clinical case studies of PEG-fusion repair of larger mixed nerves, like the sciatic, have yet to be reported; however, given the topographic organization in human peripheral nerves, it is likely that PEG-fusion repair of these nerves would show similar enhanced recovery as seen in rats (Ghergherehchi et al., 2019).

In conclusion, the presence of appropriately fused TA motor axons after PEG-fusion repair we previously reported (Ghergherehchi et al., 2019) is likely due to the topographic organization of TA axons within the sciatic nerve. This realignment of appropriate populations of axons could have contributed to the enhanced behavioral recovery seen in PEG-fused animals compared to those who received nerve injury without PEG-fusion repair. Based on our results, future work using PEG-fusion repair should prioritize realignment of territories in order to promote better behavioral recovery.

## Data Availability Statement

The original contributions presented in the study are included in the article; further inquiries can be directed to the corresponding author.

## Ethics Statement

The animal study was reviewed and approved by Bloomington Institutional Animal Care and Use Committee.

## Author Contributions

EH: study concept, experiments, data analysis, and first draft of manuscript. DS: experiments and manuscript. Both authors contributed to the article and approved the submitted version.

## Conflict of Interest

The authors declare that the research was conducted in the absence of any commercial or financial relationships that could be construed as a potential conflict of interest.

## Publisher’s Note

All claims expressed in this article are solely those of the authors and do not necessarily represent those of their affiliated organizations, or those of the publisher, the editors and the reviewers. Any product that may be evaluated in this article, or claim that may be made by its manufacturer, is not guaranteed or endorsed by the publisher.
